# Supplementary figures and images for: HLJ1 amplifies endotoxin-induced sepsis severity by promoting IL-12 heterodimerization in macrophages
Source: eLife. 2022 Aug 19;11:e76094. doi: 10.7554/eLife.76094 (PMC9457701; doi:10.7554/eLife.76094)

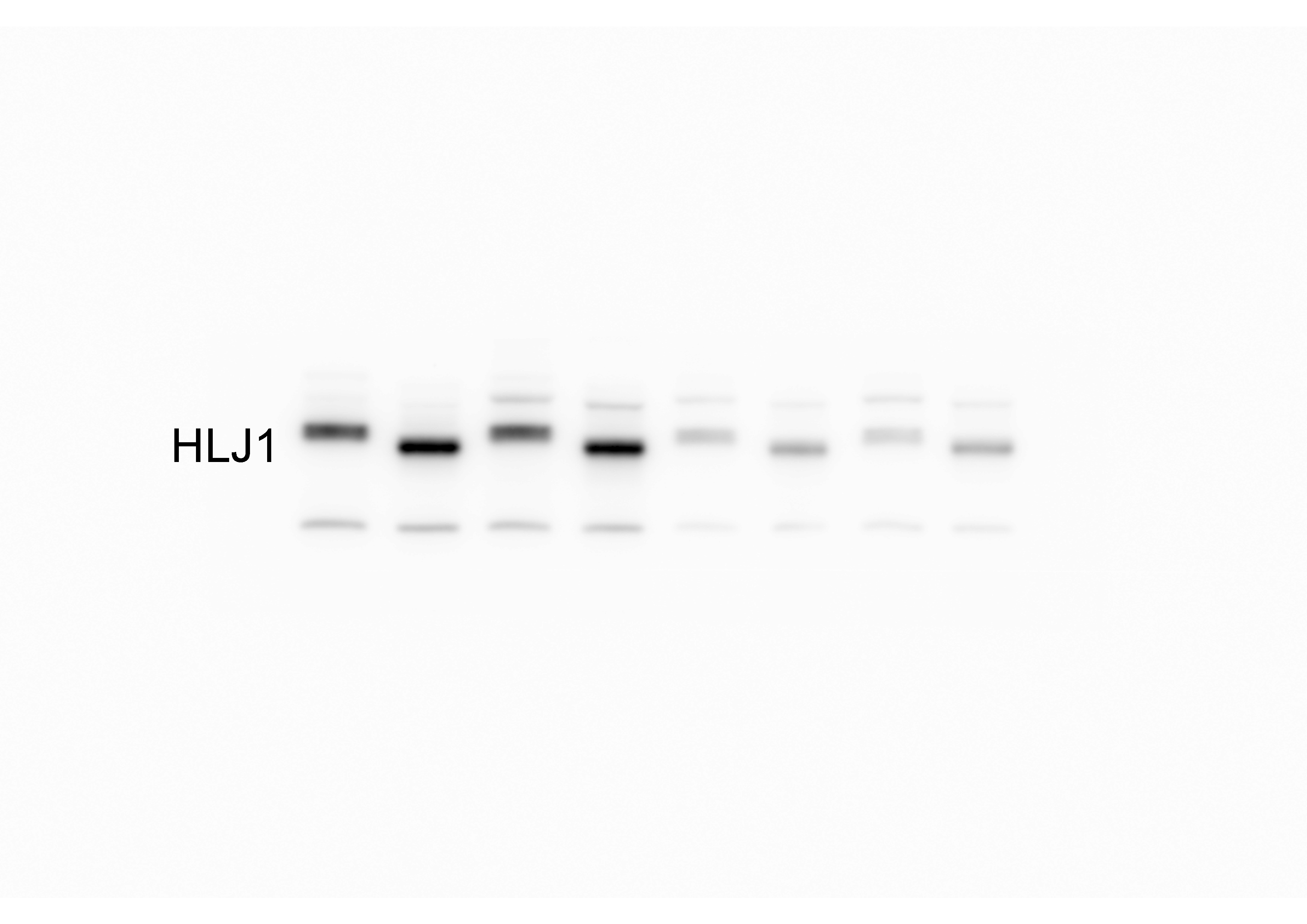

Supplement: Figure 9—source data 2. [file elife-76094-fig9-data2.zip › Figure 9- source data 2/Figure 9E/labelled_blots/HLJ1_labelled.tif]

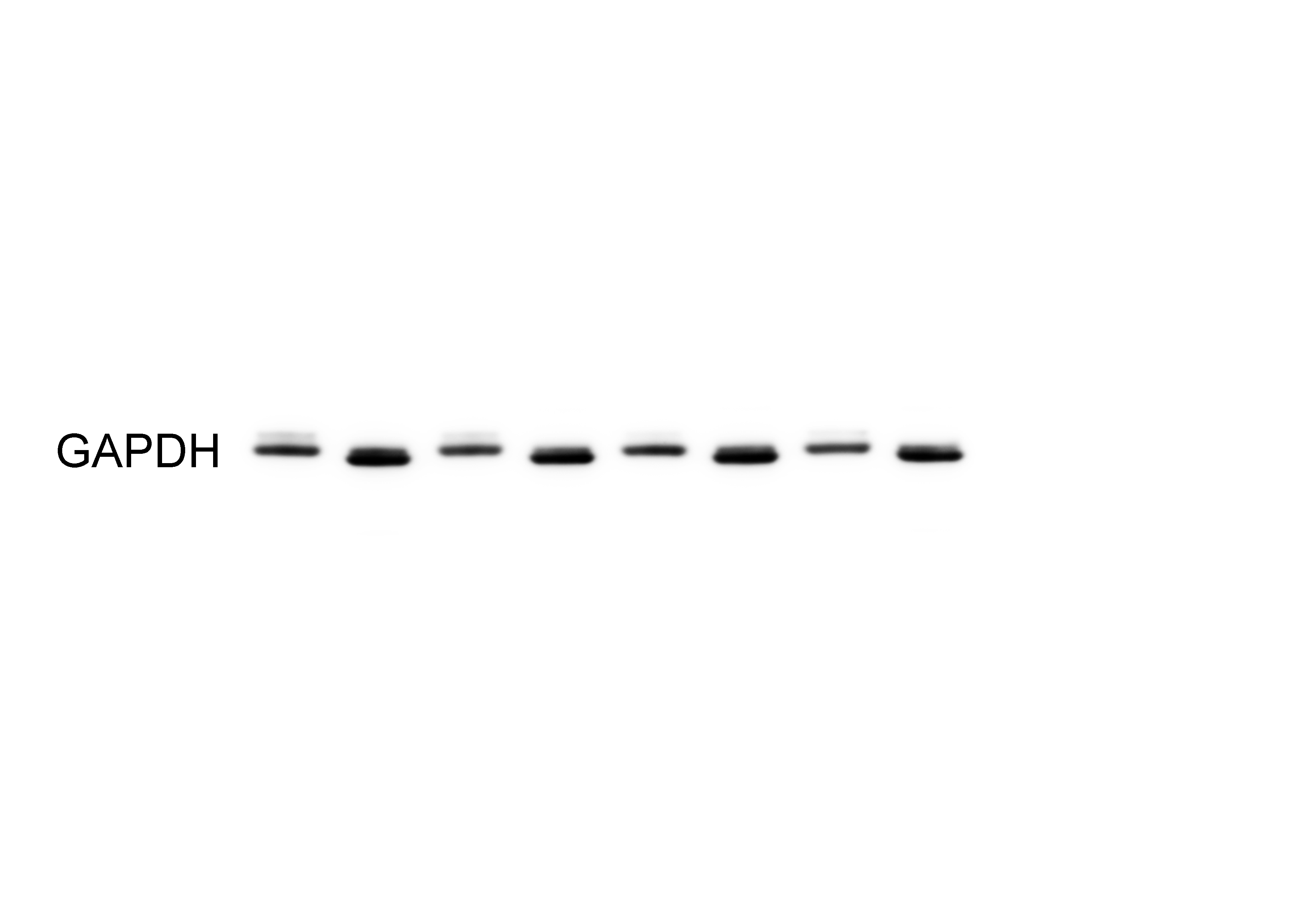

Supplement: Figure 9—source data 2. [file elife-76094-fig9-data2.zip › Figure 9- source data 2/Figure 9E/labelled_blots/GAPDH_labelled.tif]

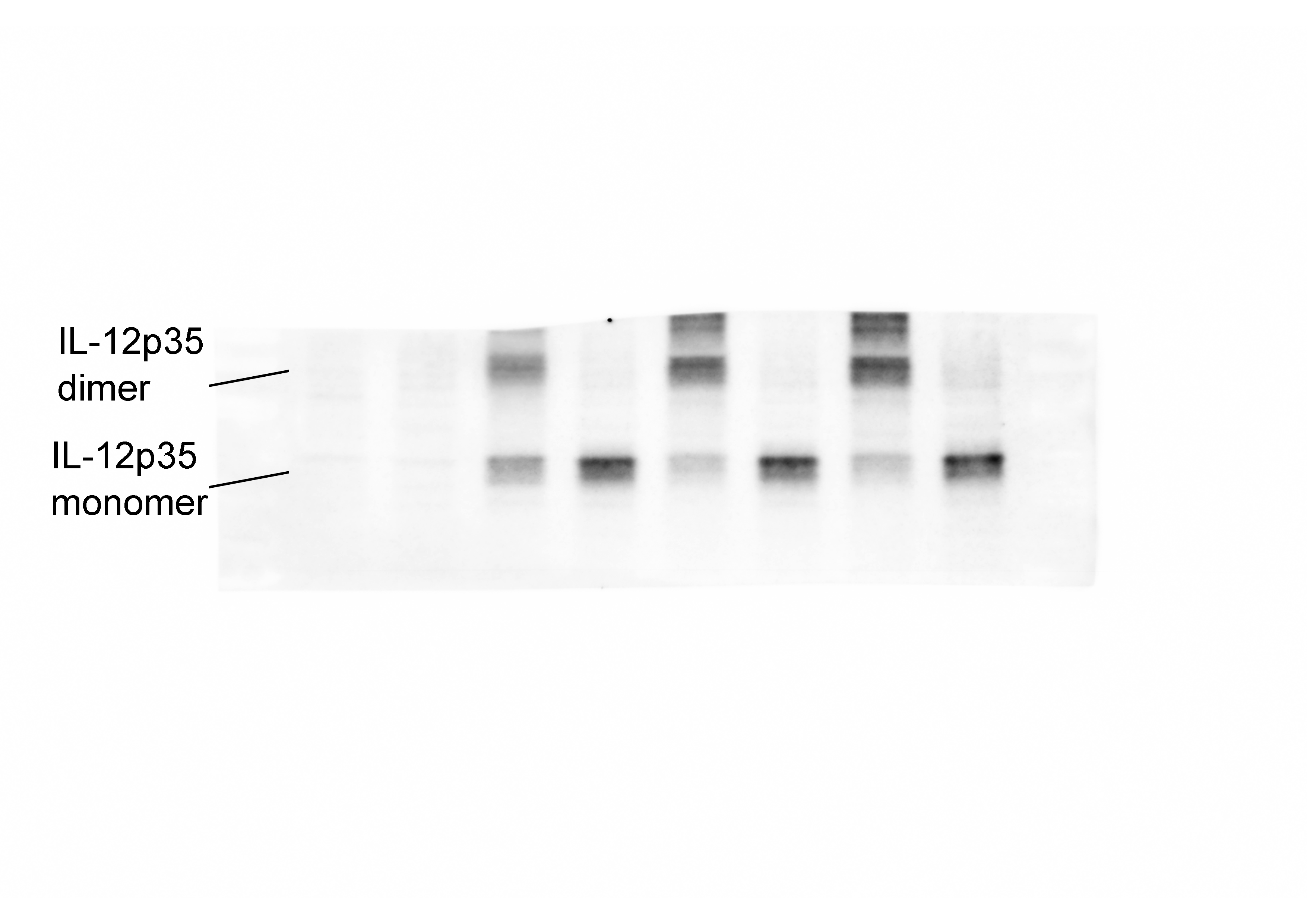

Supplement: Figure 9—source data 2. [file elife-76094-fig9-data2.zip › Figure 9- source data 2/Figure 9E/labelled_blots/Il12p35_labelled.tif]

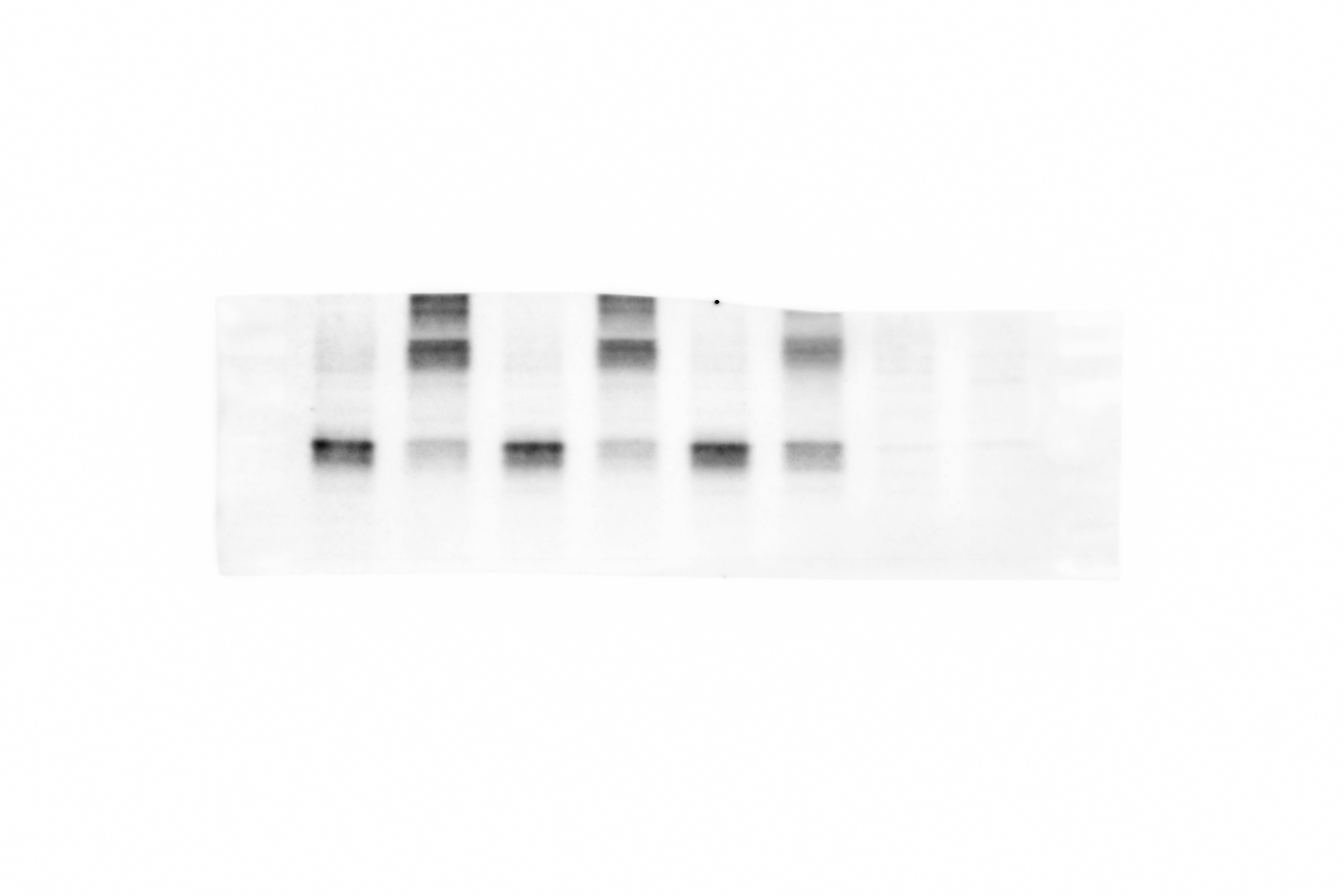

Supplement: Figure 9—source data 2. [file elife-76094-fig9-data2.zip › Figure 9- source data 2/Figure 9E/original_blots/IL12p35_original.tif]

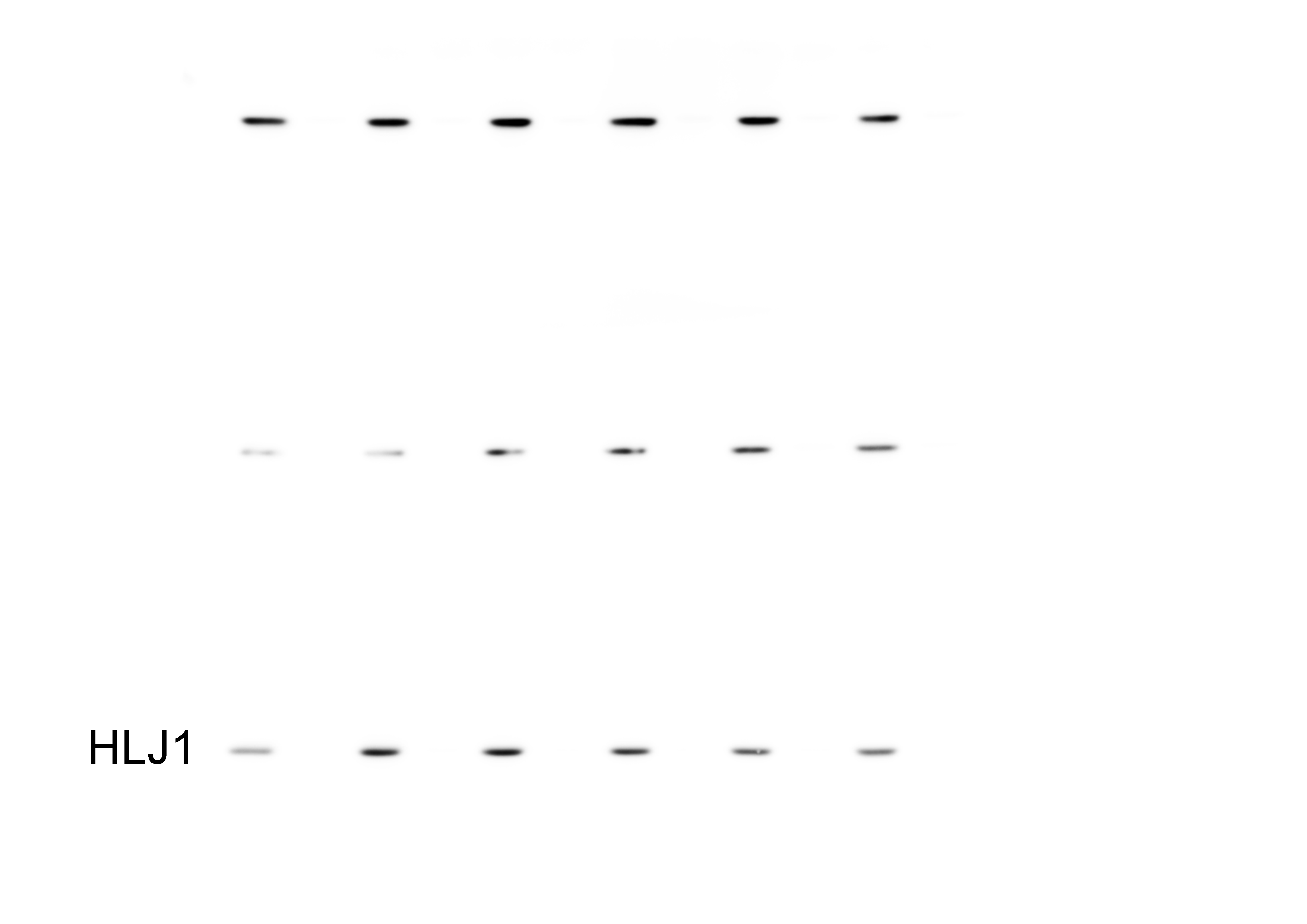

Supplement: Figure 9—source data 2. [file elife-76094-fig9-data2.zip › Figure 9- source data 2/Figure 9D/labelled_blot/HLJ1_labelled.tif]

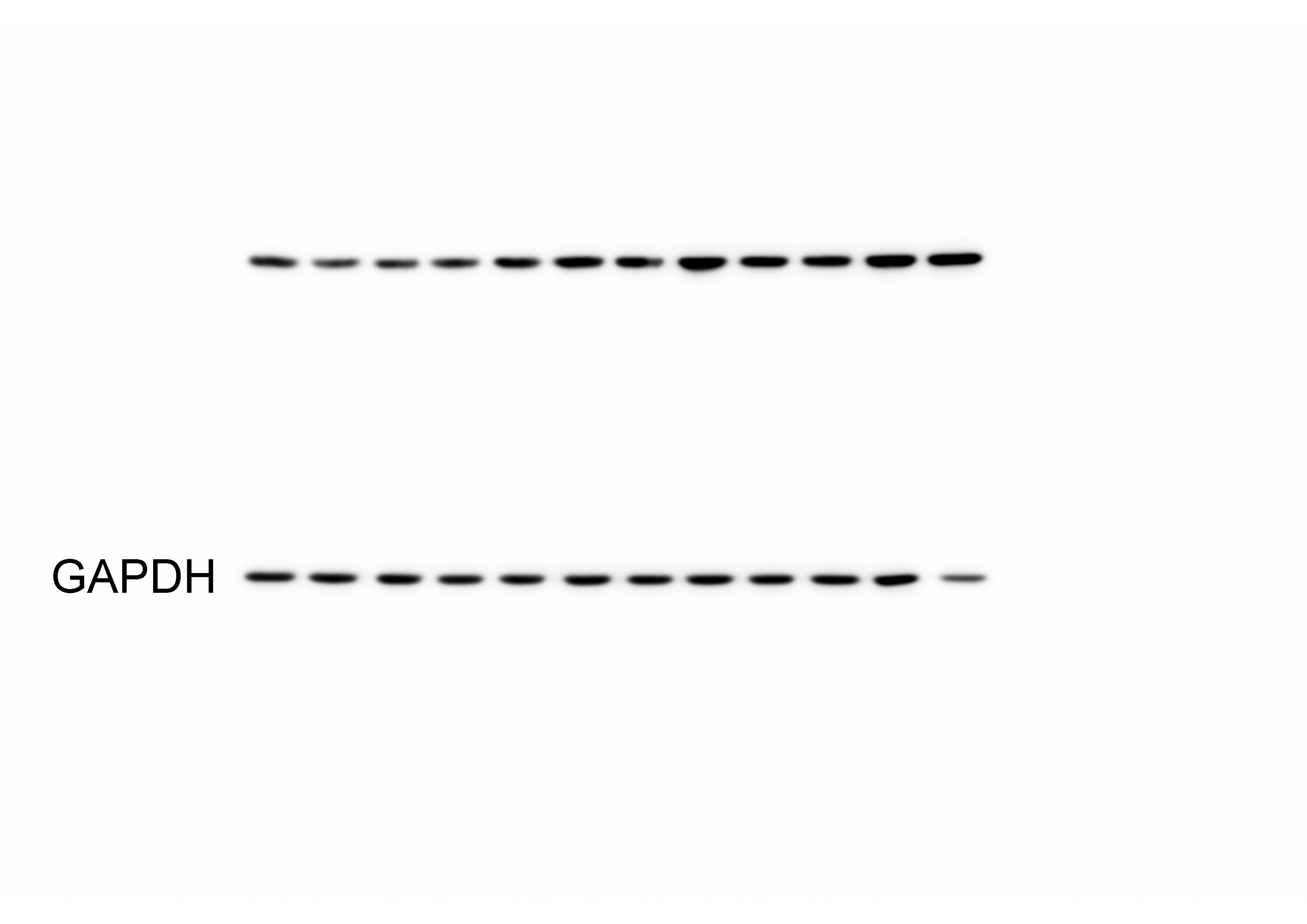

Supplement: Figure 9—source data 2. [file elife-76094-fig9-data2.zip › Figure 9- source data 2/Figure 9D/labelled_blot/GAPDH_labelled.tif]

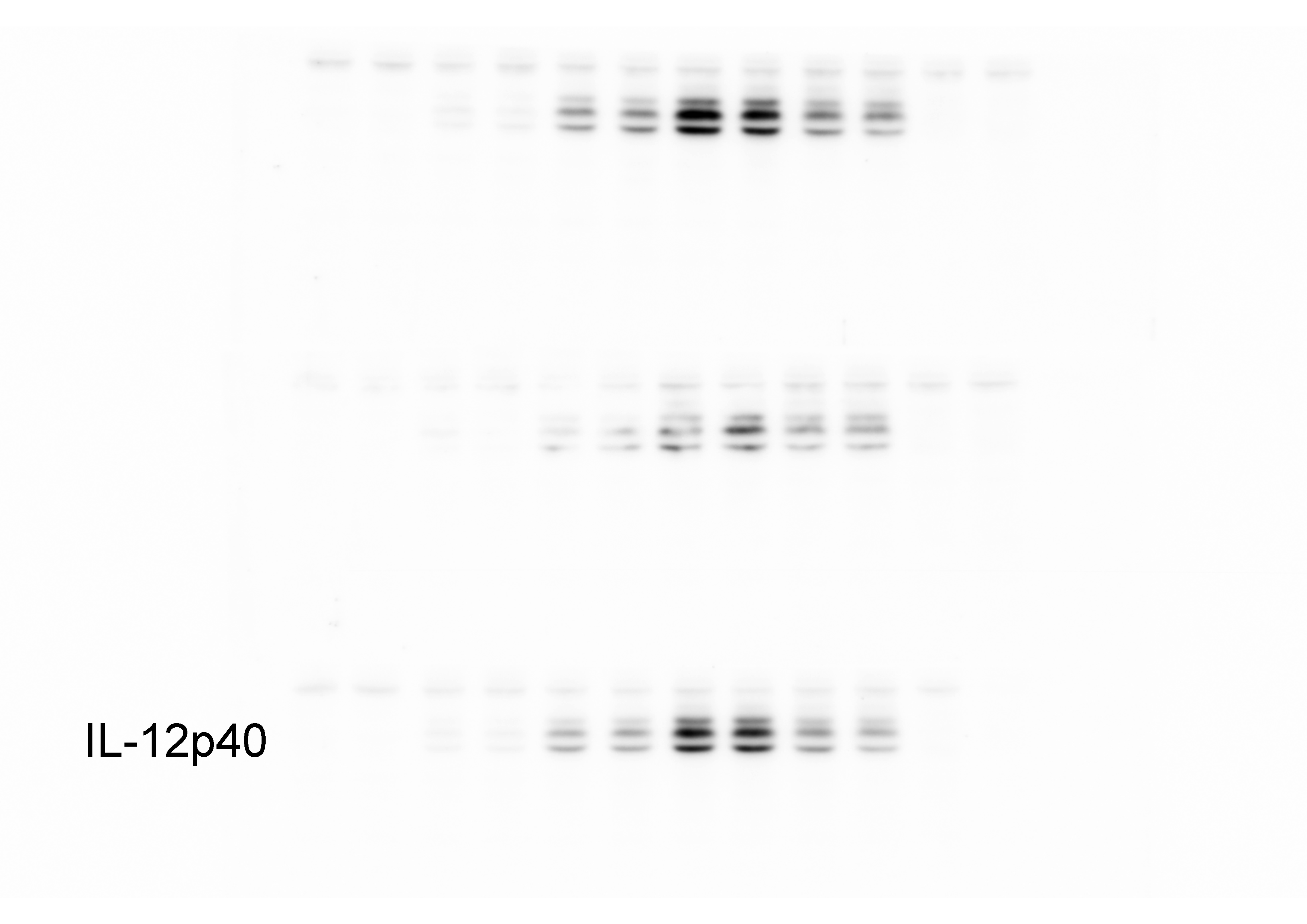

Supplement: Figure 9—source data 2. [file elife-76094-fig9-data2.zip › Figure 9- source data 2/Figure 9D/labelled_blot/IL12p40_labelled.tif]

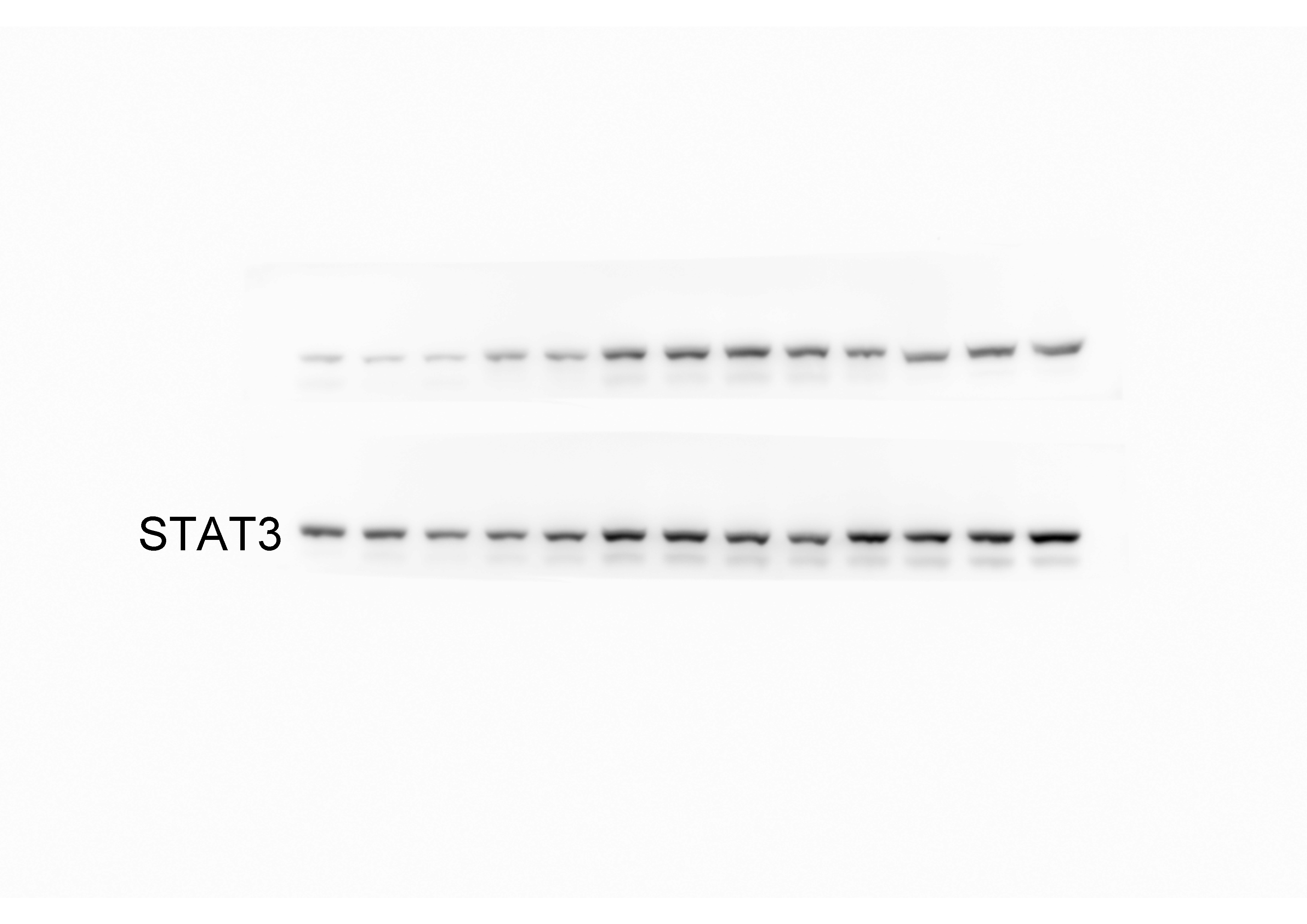

Supplement: Figure 9—figure supplement 2—source data 1. [file elife-76094-fig9-figsupp2-data1.zip › Figure 9- figure supplement 2- source data 2/Figure 9-figure supplement 2A/labelled blots/STAT3_labelled.tif]

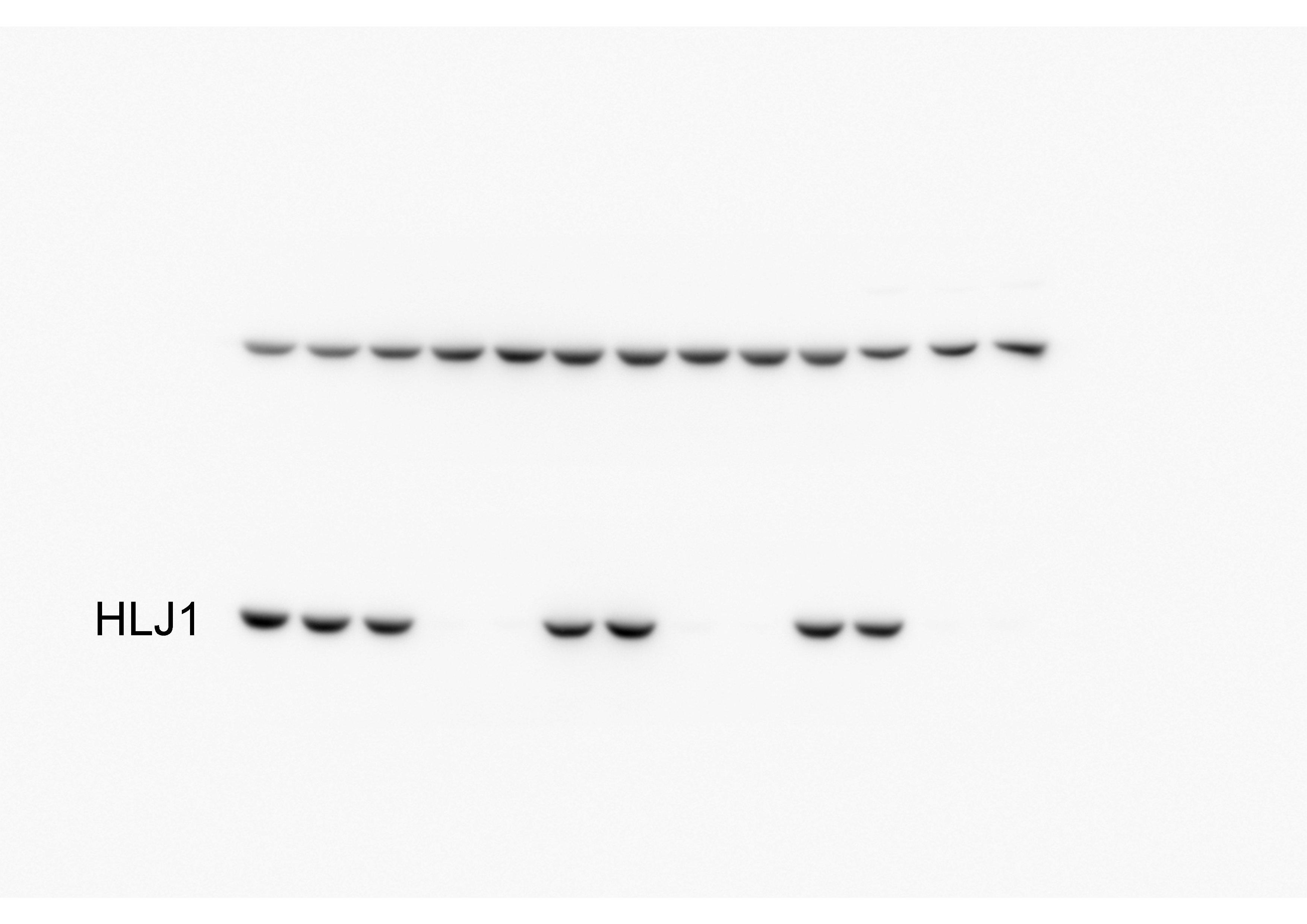

Supplement: Figure 9—figure supplement 2—source data 1. [file elife-76094-fig9-figsupp2-data1.zip › Figure 9- figure supplement 2- source data 2/Figure 9-figure supplement 2A/labelled blots/HLJ1_labelled.tif]

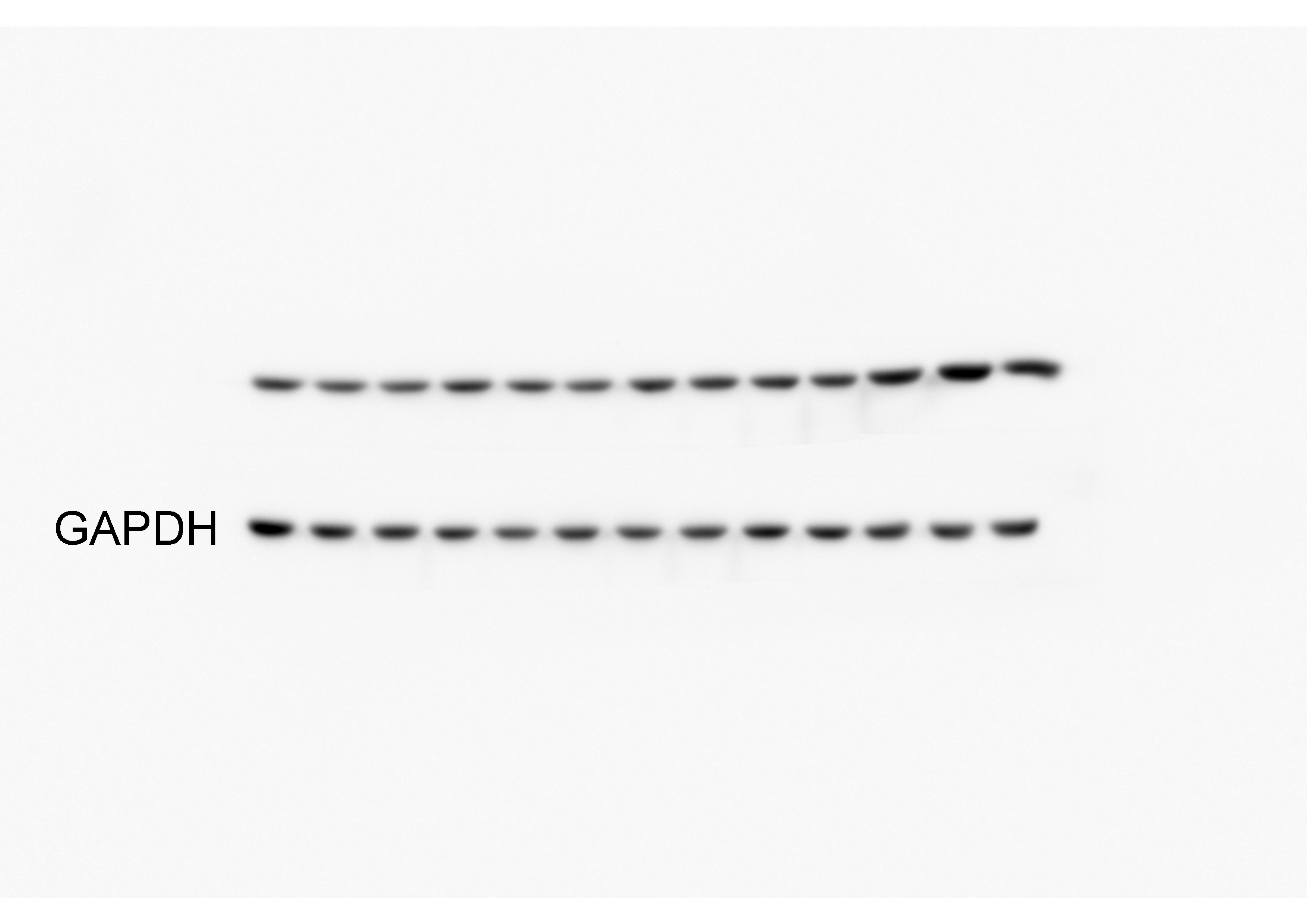

Supplement: Figure 9—figure supplement 2—source data 1. [file elife-76094-fig9-figsupp2-data1.zip › Figure 9- figure supplement 2- source data 2/Figure 9-figure supplement 2A/labelled blots/GAPDH_labelled.tif]

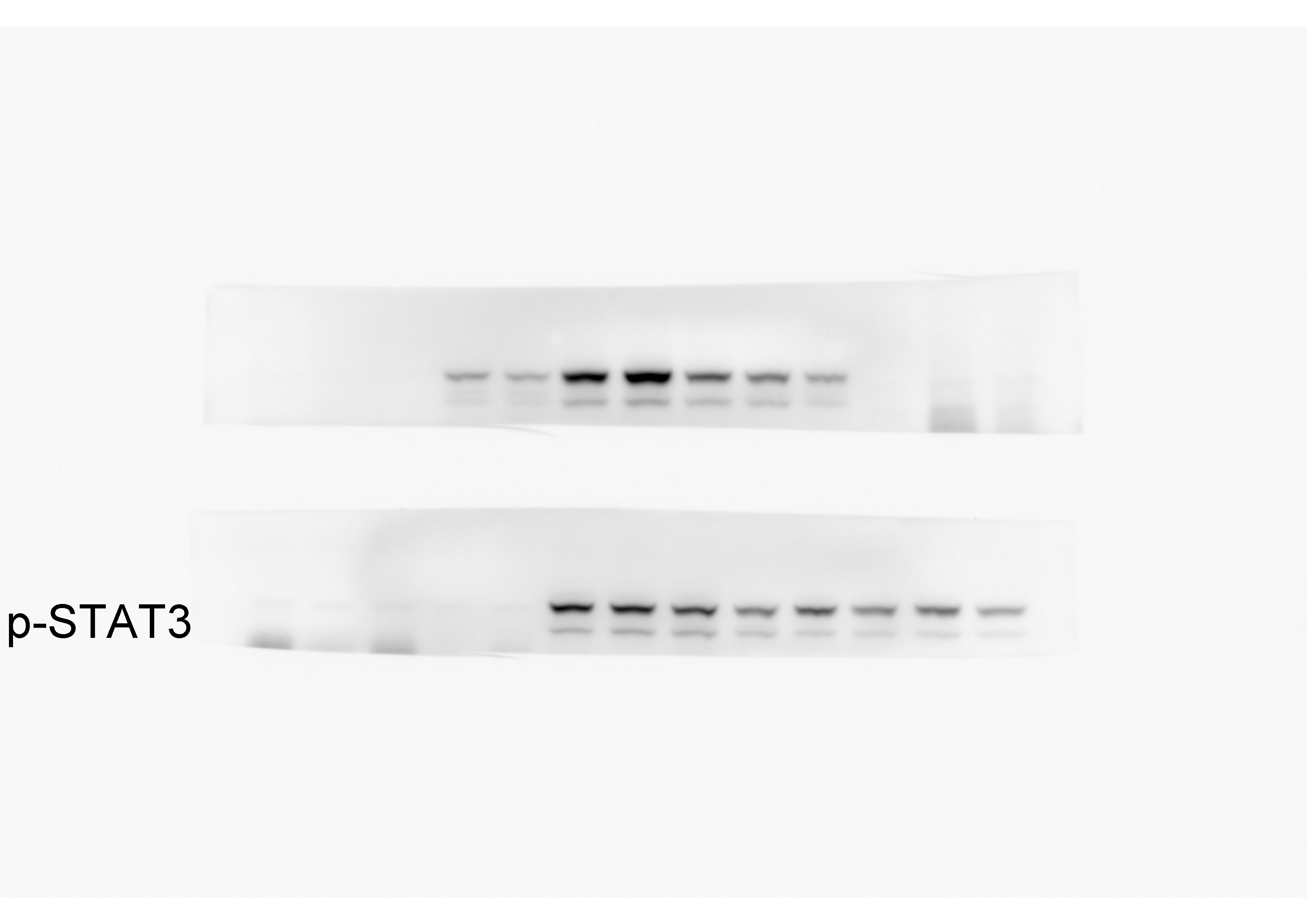

Supplement: Figure 9—figure supplement 2—source data 1. [file elife-76094-fig9-figsupp2-data1.zip › Figure 9- figure supplement 2- source data 2/Figure 9-figure supplement 2A/labelled blots/p-STAT3_labelled.tif]

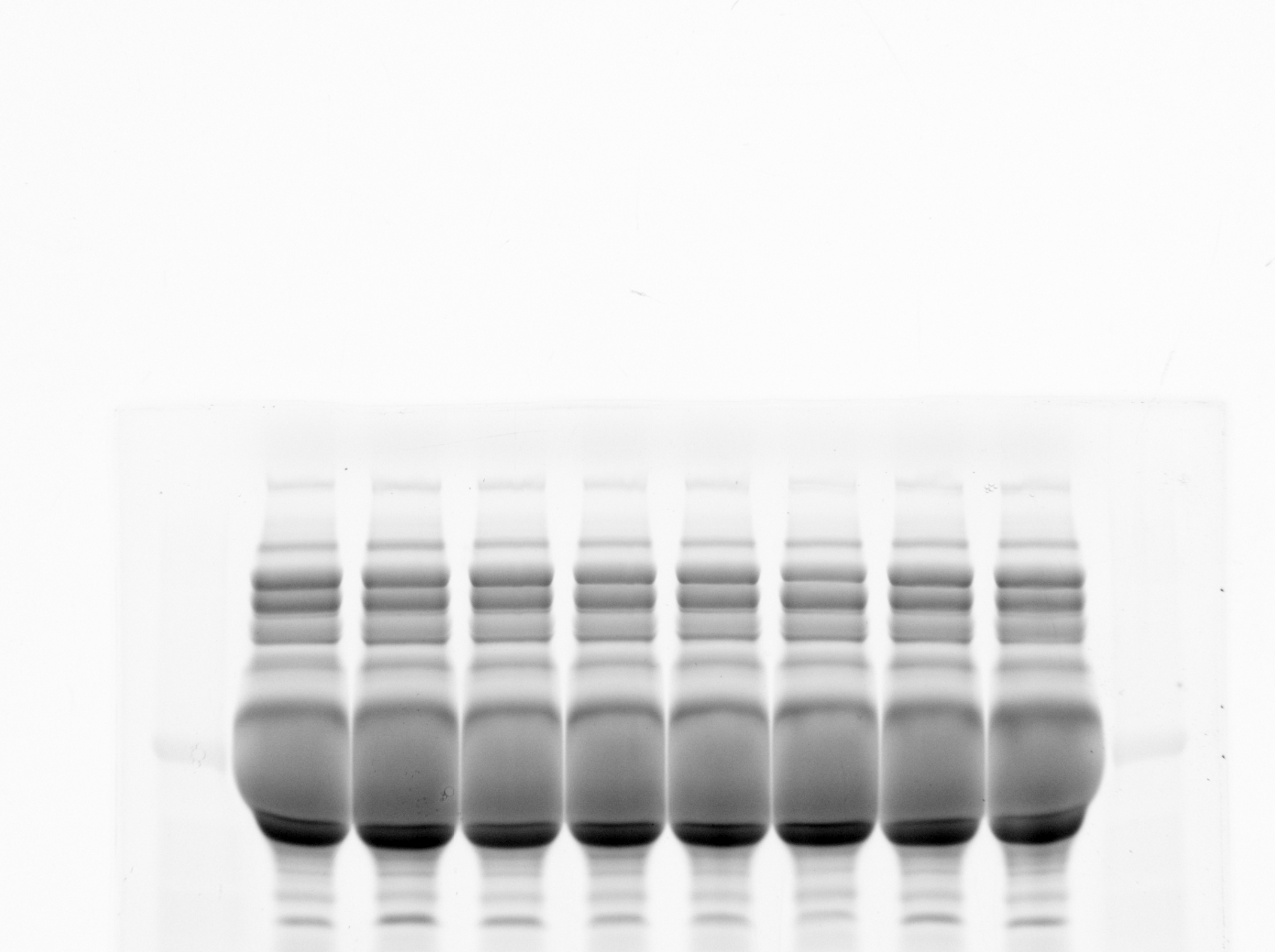

Supplement: Figure 9—figure supplement 2—source data 1. [file elife-76094-fig9-figsupp2-data1.zip › Figure 9- figure supplement 2- source data 2/Figure 9-figure supplement 2C/original/total protein right panel.tif]

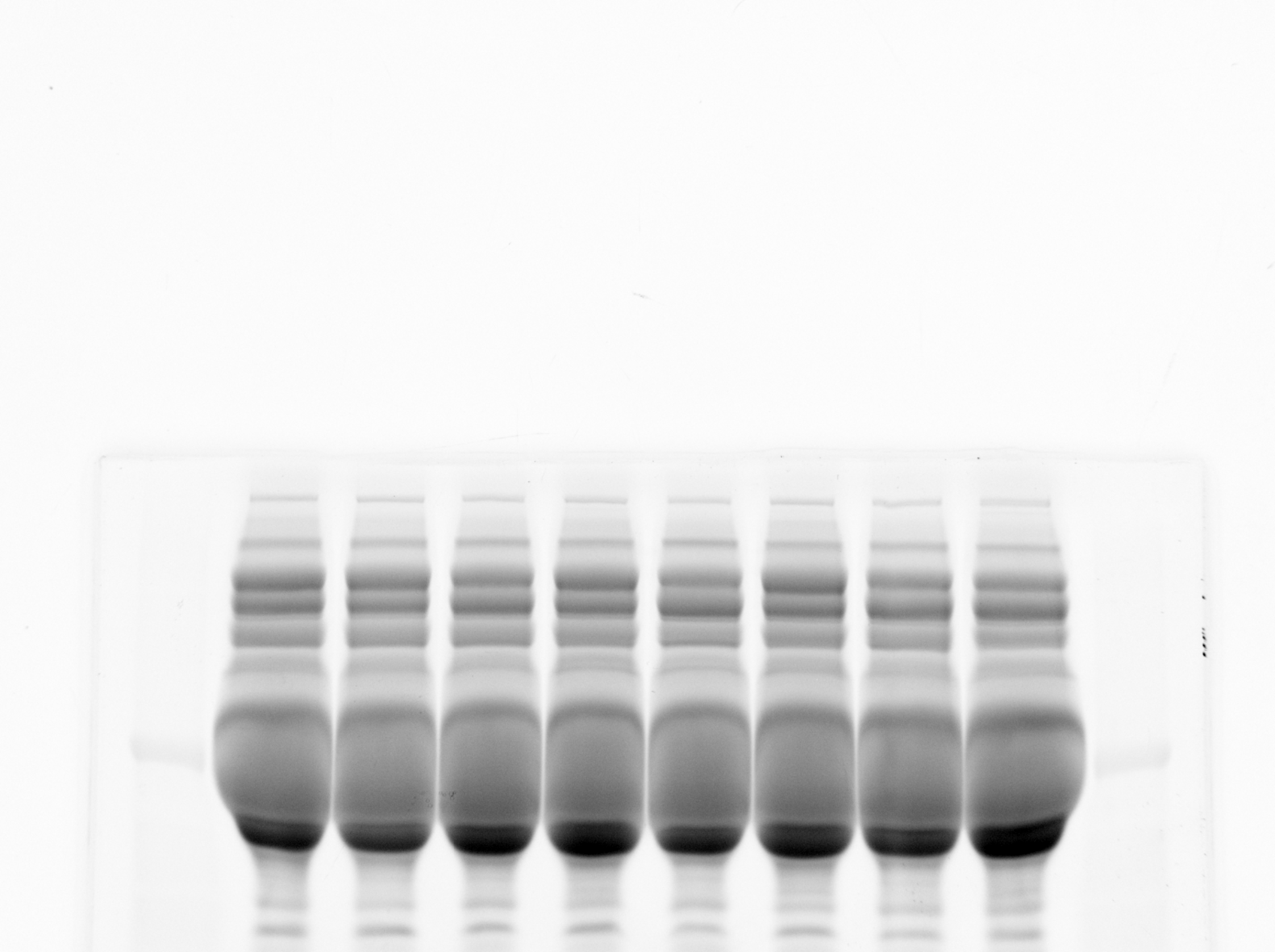

Supplement: Figure 9—figure supplement 2—source data 1. [file elife-76094-fig9-figsupp2-data1.zip › Figure 9- figure supplement 2- source data 2/Figure 9-figure supplement 2C/original/total protein left panel.tif]

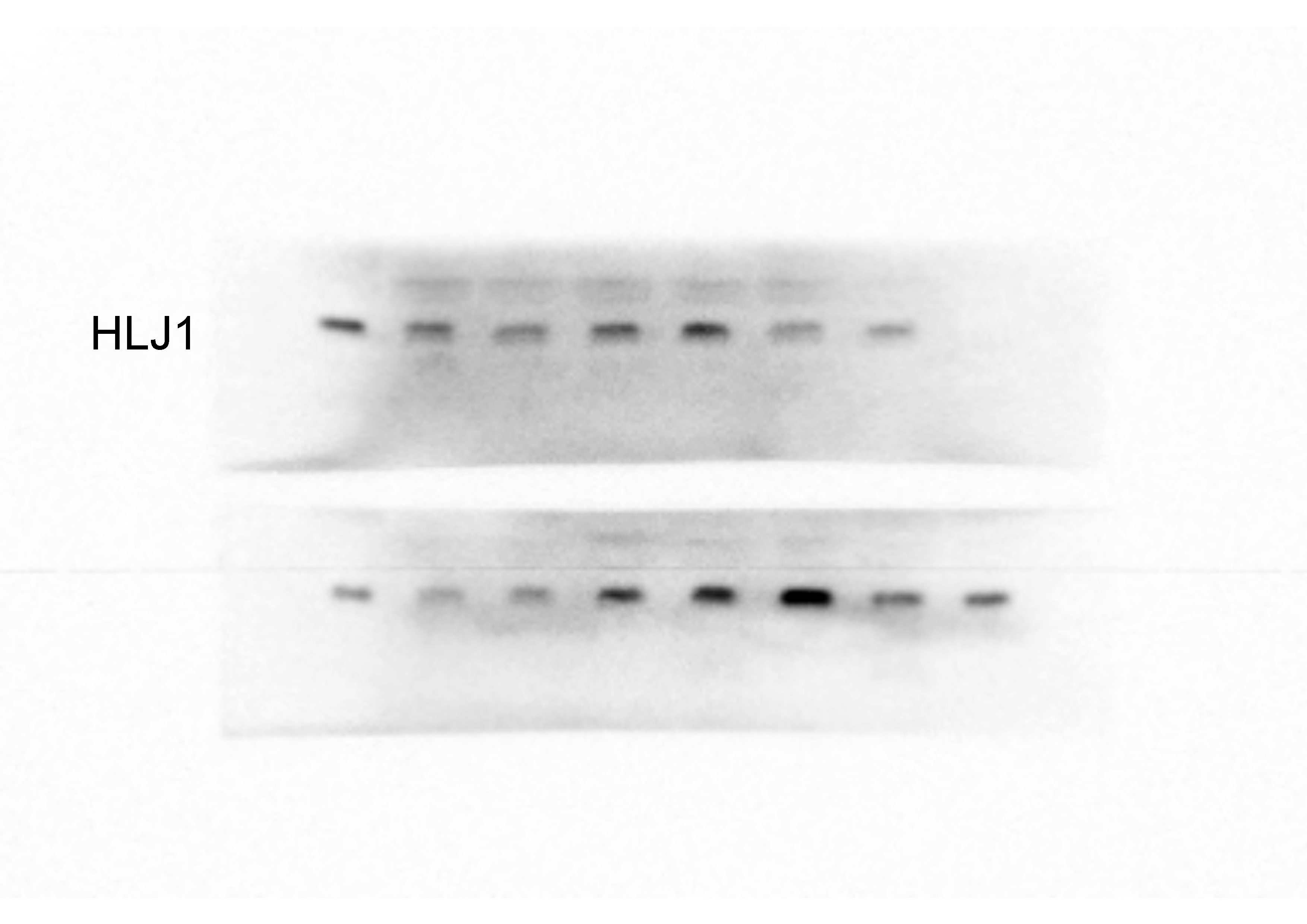

Supplement: Figure 9—figure supplement 2—source data 1. [file elife-76094-fig9-figsupp2-data1.zip › Figure 9- figure supplement 2- source data 2/Figure 9-figure supplement 2C/labelled/HLJ1 right panel_labelled.tif]

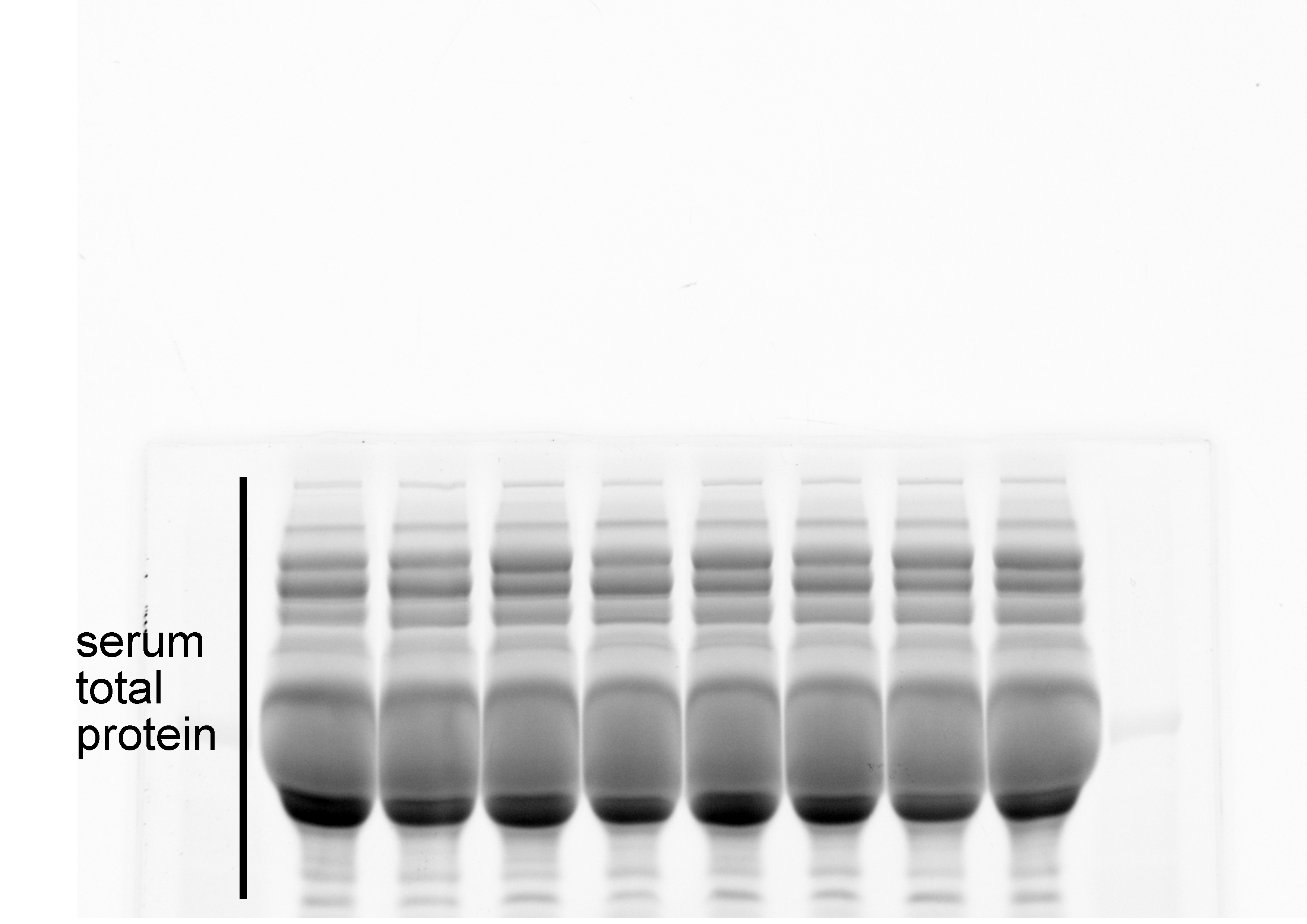

Supplement: Figure 9—figure supplement 2—source data 1. [file elife-76094-fig9-figsupp2-data1.zip › Figure 9- figure supplement 2- source data 2/Figure 9-figure supplement 2C/labelled/total protein left panel_labelled.tif]

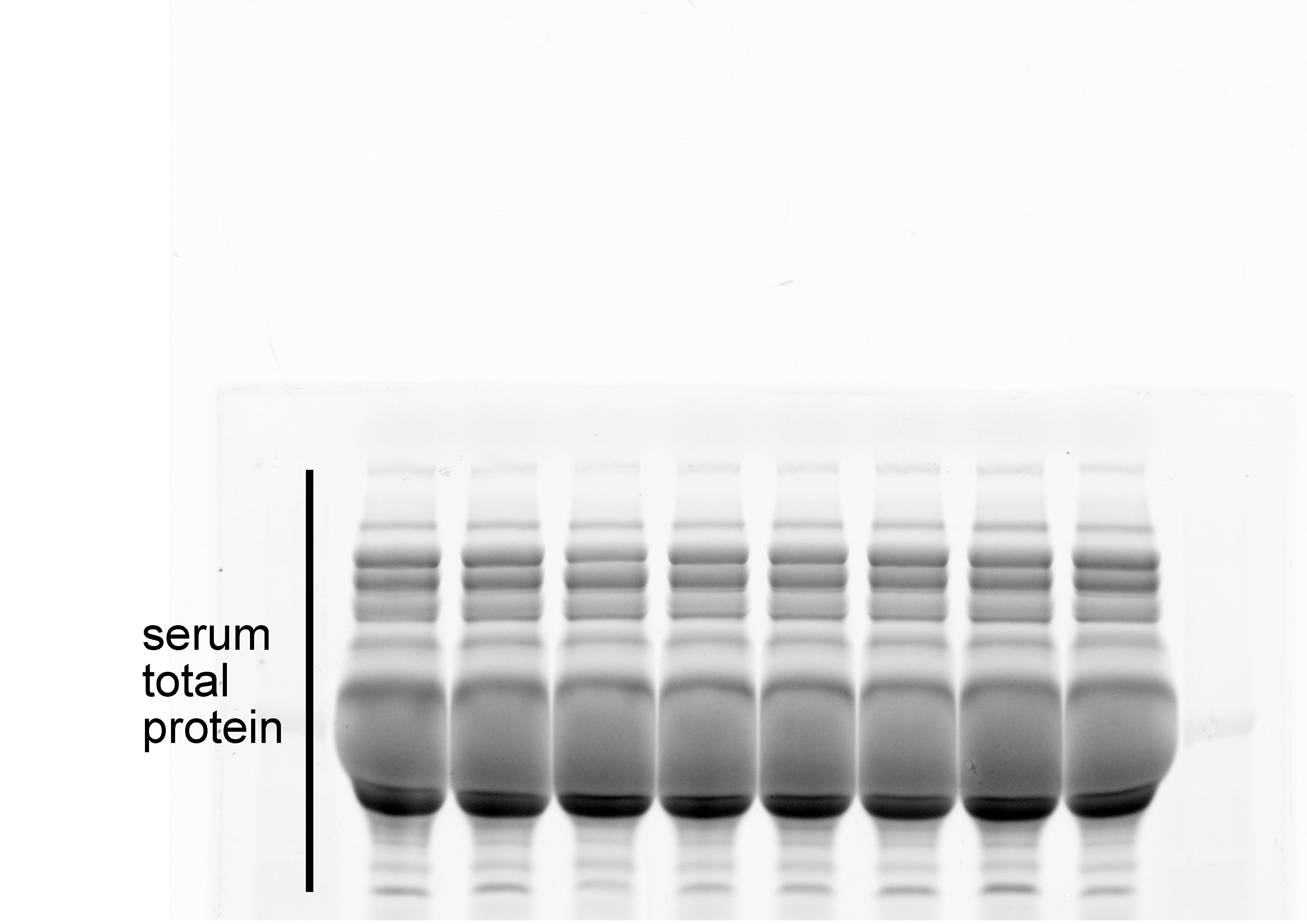

Supplement: Figure 9—figure supplement 2—source data 1. [file elife-76094-fig9-figsupp2-data1.zip › Figure 9- figure supplement 2- source data 2/Figure 9-figure supplement 2C/labelled/total protein right panel_labelled.tif]

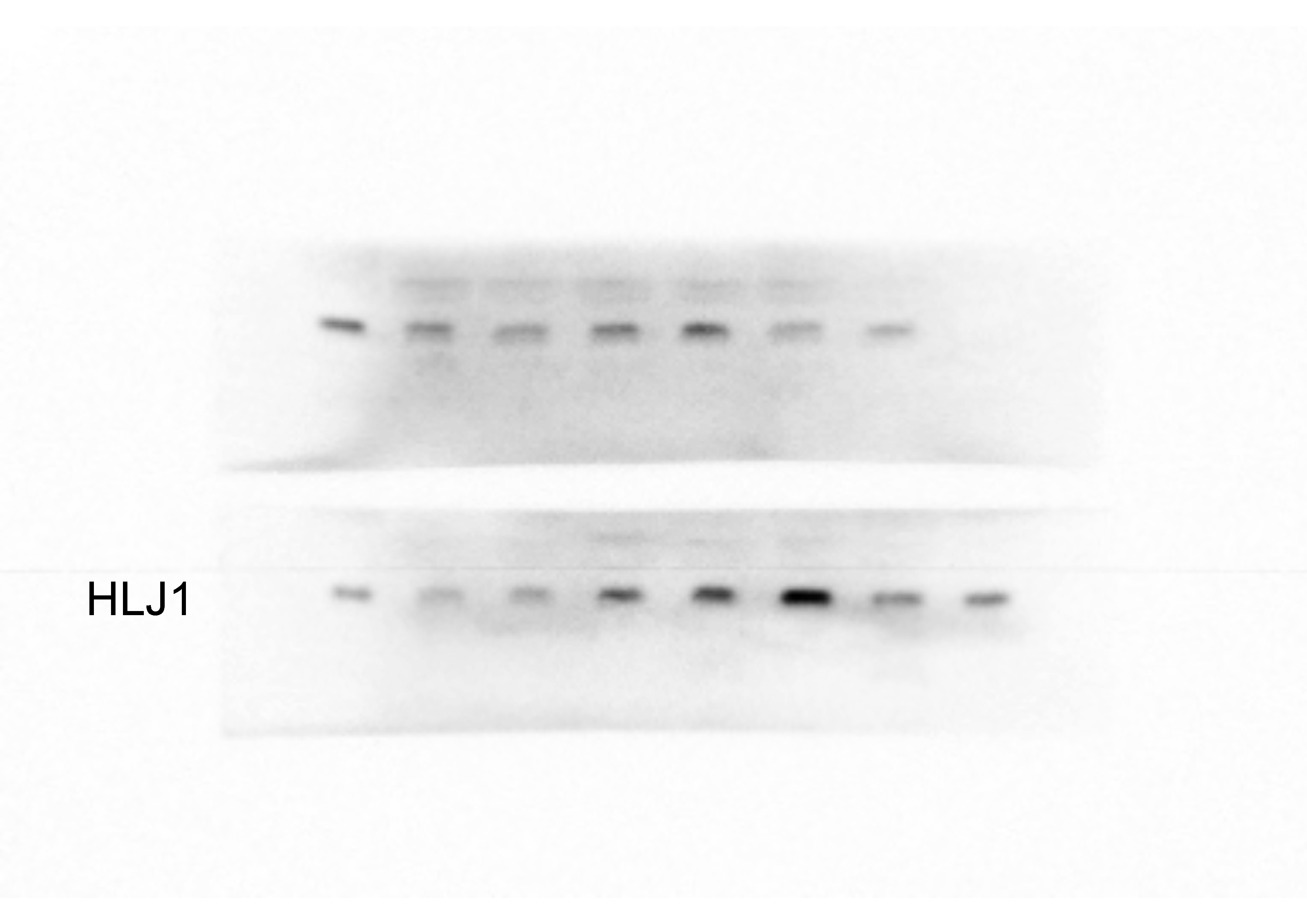

Supplement: Figure 9—figure supplement 2—source data 1. [file elife-76094-fig9-figsupp2-data1.zip › Figure 9- figure supplement 2- source data 2/Figure 9-figure supplement 2C/labelled/HLJ1 left panel_labelled.tif]

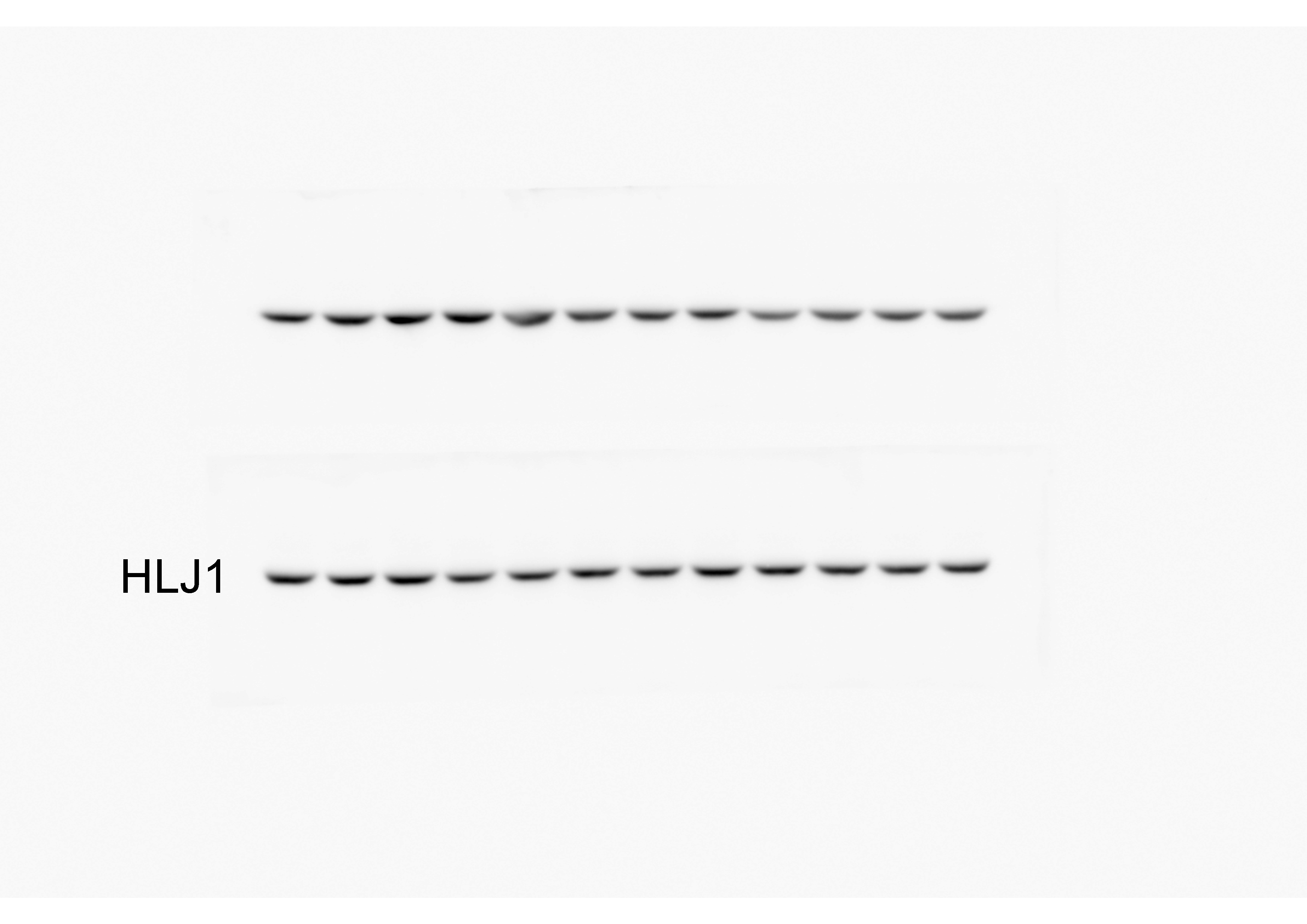

Supplement: Figure 9—figure supplement 2—source data 1. [file elife-76094-fig9-figsupp2-data1.zip › Figure 9- figure supplement 2- source data 2/Figure 9-figure supplement 2B/labelled blots/HLJ1_labelled.tif]

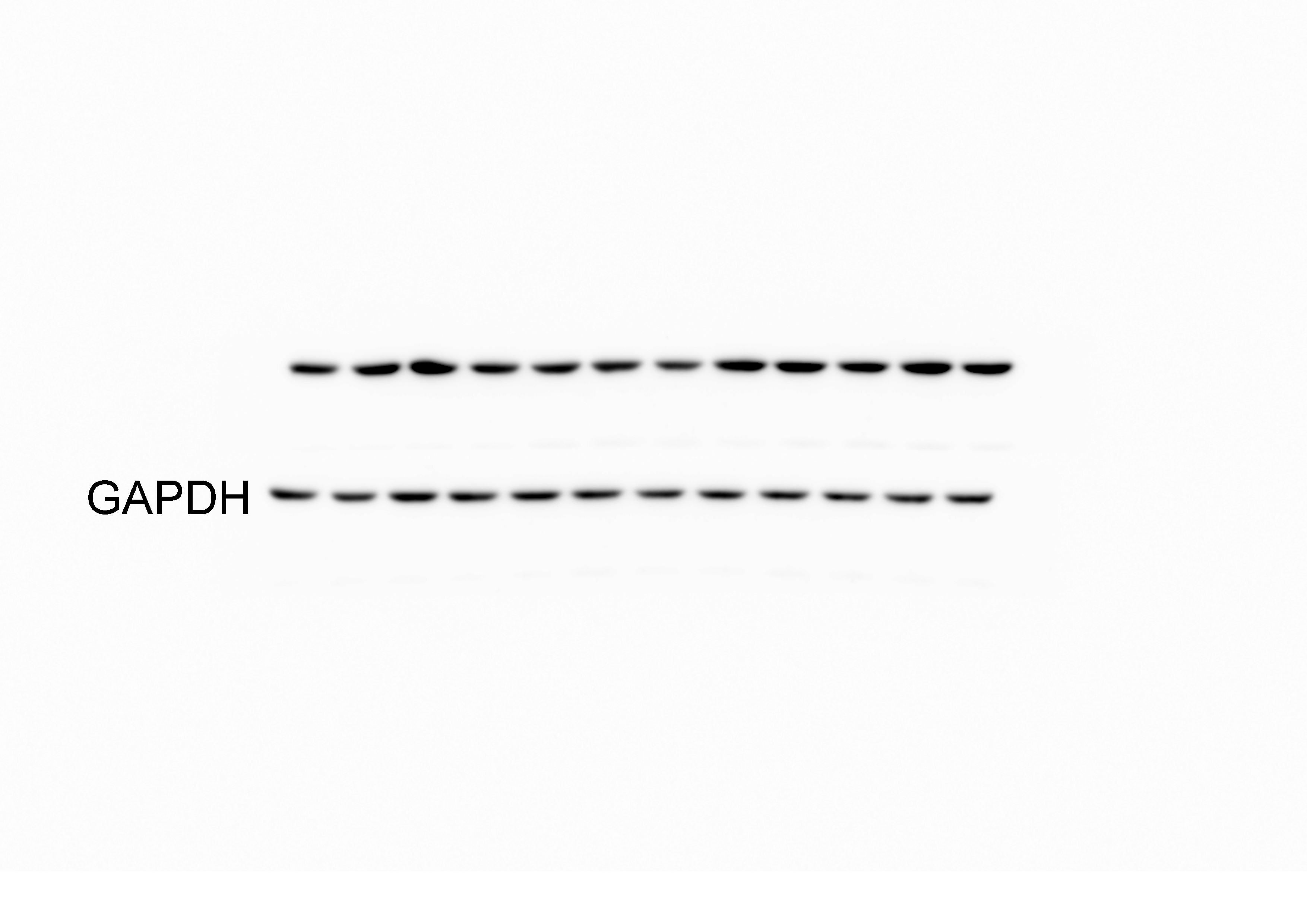

Supplement: Figure 9—figure supplement 2—source data 1. [file elife-76094-fig9-figsupp2-data1.zip › Figure 9- figure supplement 2- source data 2/Figure 9-figure supplement 2B/labelled blots/GAPDH_labelled.tif]
